# Public understanding of female genital anatomy and pelvic organ prolapse (POP); a questionnaire-based pilot study

**DOI:** 10.1007/s00192-021-04727-9

**Published:** 2021-03-31

**Authors:** Dina El-Hamamsy, Chanel Parmar, Stephanie Shoop-Worrall, Fiona M. Reid

**Affiliations:** 1 The Warrell Unit, Saint Mary’s Hospital, Manchester Academic Health Science Centre, Manchester University NHS Foundation Trust, Hathersage Road, Manchester, M13 9WL UK; 2 5th year medical student, Manchester University, Manchester, UK; 3 Centre for Health Informatics, Manchester Academic Health Sciences Centre, The University of Manchester, Manchester, M13 9PT UK; 4 Centre for Epidemiology Versus Arthritis, Manchester Academic Health Sciences Centre, The University of Manchester, Manchester, M13 9PT UK; 5 Institute of Human Development, Faculty of Medical & Human Sciences, Manchester Academic Health Science Centre, University of Manchester, Oxford Road, Manchester, M13 9PL UK

**Keywords:** Pelvic organ prolapse, Female genital anatomy, Public understanding, Shared decision making, Gynaecology, Health literacy

## Abstract

**Introduction and hypothesis:**

Health literacy underpins informed consent and shared decision-making. In gynaecology, this includes understanding of normal anatomy and urogenital disease. This study evaluated public knowledge of external female genital anatomy and pelvic organ prolapse (POP).

**Methods:**

A questionnaire study asked participants for their demographics and to label a female external genitalia diagram and included free-text questions on POP, its symptoms and treatment. Questionnaires were distributed at general outpatient (OPD) and urogynaecology (UG) departments at a UK teaching hospital. Differences in the number of correct anatomy labels between participant genders were assessed via chi-squared tests and, within female participants, multivariable linear and logistic regressions assessed associations with increasing correct anatomical labels and an understanding (versus no understanding) of POP, respectively.

**Results:**

Within 191 (*n* = 160 OPD, *n* = 31 UG), 9/103 (9%) labelled all anatomical structures correctly. Females had more correct labels (median 1, IQR 0,3) versus males (median 0, IQR 0,1), *P* = 0.022). Higher education (vs. < secondary) and white ethnicity were associated with greater numbers of correct labels [coefficient (95% CI): 1.05 (0.14, 1.96), *P* = 0.024, 1.45 (0.58, 2.33), *P* = 0.001 respectively]. Fifty-three per cent understood POP. POP understanding increased with increasing age, white ethnicity (OR: 4.38, 95% CI: 1.36, 14.08, *P* = 0.013) and more correct anatomy labels (OR: 1.43, 95% CI 1.14, 1.79, *P* = 0.002). Of those who understood POP, only 35% identified “bulge” as a symptom and 7% physiotherapy as a treatment option.

**Conclusion:**

There was poor public understanding of external female genital anatomy and POP, which may have significant implications for health-seeking, shared decision-making and informed consent.

**Supplementary Information:**

The online version contains supplementary material available at 10.1007/s00192-021-04727-9.

## Introduction

Health literacy is defined as “the extent to which people are able to understand and act upon health information” [1]. It has been shown to affect health-seeking behaviour, engagement with treatment, preparedness for surgery, patient satisfaction and litigation [2–6]. Specifically in surgical patients, poor health literacy has been shown to reduce adherence to perioperative instructions and is associated with poor outcomes [7].

The recent Independent Medicines and Medical Devices Safety Review (Cumberlege Report) included extensive investigation of healthcare provision for women affected by mesh complications in the UK. One of the key themes highlighted in this report was failure of informed consent [1, 5]. A key factor of informed consent is understanding of the condition and also the normal anatomy and physiology.

Concerns have repeatedly been raised that women do not receive adequate information, counselling and choice prior to surgical intervention for pelvic organ prolapse (POP) [8–10]. POP has been described as a “disease of shame and silence” in which many women feel uncomfortable discussing their condition with friends, family and also healthcare professionals [11, 12]. If women are to participate in shared decision-making for POP interventions, there first needs to be a basic understanding of normal anatomy and pathophysiology of the disease process. Furthermore, complex treatment options must be discussed at an understandable level to facilitate discussion. However, the level of health literacy regarding healthy female genital anatomy in the general population is unclear, and approximately half of self-reported health-related quality of life questionnaires of pelvic floor dysfunction (PFD) are above the advised readability levels [13]. In an effort to improve patient understanding, the National Institute for Health and Care Excellence (NICE) also published Decision Aids to help patients decide which prolapse or incontinence treatment options best suit them as individuals [14–16]. However, these do not address basic anatomical structures.

Women can have poor understanding of PFD despite high health literacy scores [17]. Women are also less likely to understand the actual condition of POP than the possible treatments available [12]. It has been suggested that this is because more time is spent, by healthcare professionals, discussing the treatment rather than the condition itself [12] or due to patients’ struggle to grasp certain diagnostic terms [18]. However, it may also be due to poor background understanding of female genital anatomy among the public in general.

In this study we aimed to examine the general public’s knowledge of normal external female genital anatomy and their understanding of pelvic organ prolapse in comparison to their understanding of other general medical or gynaecological conditions. We hypothesised that males may understand less about these topics and thus included them in our study.

A secondary aim was to compare such knowledge and understanding between those attending a general outpatients department (OPD) (males and females) and specialist urogynaecology (UG) clinics (females only).

## Methods

### Ethical approval

The study was approved by the North West Ethics committee, REC reference: 17/NW/0215, IRAS project ID: 206362.

### Study population

One hundred ninety-one anonymous questionnaires were distributed by the researcher (CP) throughout the general OPD and UG clinic of a tertiary UK hospital. As the study was aimed at investigating the general public’s understanding of the topic, both patients and their companions, e.g. family, friends or carers, were invited to participate in the study. Male participants were recruited from the general OPD. Participants were approached while awaiting clinic appointments, after gaining permission from clinical staff. This ensured that patients’ clinical care was not compromised by discussing the study with the researcher or filling the questionnaire at that time. Verbal consent was obtained from all participants.

Purposive sampling was used to obtain an evenly age-distributed sample from both UG (females only) and OPD (males and females), including male participants. As the study aimed primarily to capture the general public’s understanding of the topic, questionnaires were mainly distributed in the OPD (160 vs. 31 in UG) (Table 1). Such distribution allowed maximum variation sampling to capture a broad view of the background population knowledge [19, 20].

**Table 1:** Distribution of population sample ( upper part of table) and definitions of conditions ( lower part of table)

**Age**	**UG**	**OPD females**	**OPD males**	**Total**
18–25	1^$^	35	5	41
26–45	10	35	5	50
46–65	10	35	5	50
>65	10	35	5	50
Total	31	140	20	191
**Disease**	Definition^*^			
Diabetes	Problem with blood sugar - description of risk factors, pathophysiology, symptoms, complications/management also accepted - "low blood sugar" NOT accepted.			
Stroke	(1) Pathophysiological description - it is a condition affecting the brain and /or (2) Symptom definition it causes paralysis / loss of speech.			
Fibroid	Growth in uterus - partial understanding accepted eg problem with womb.			
Prolapse	Descent of any (pelvic) organ- description of risk factors, pathophysiology (e.g. weakness of pelvic muscles), symptoms (e.g. vaginal bulge or lump), partial understanding also accepted. Descriptions of any organ prolapse i.e. "descent of any organ" is also accepted as the question did not specify "pelvic organ prolapse".			

*POP*, pelvic organ prolapse; *OPD*, outpatient department; *UG*, Urogynaecology

^$^Only one participant was recruited in this category (as opposed to 10 initially planned, to obtain an even distribution among participants age groups) as pelvic floor dysfunction is less common among this age group, hence the total number of received questionnaires was 191. Age was measured in years

^*^Any description relating to/ variation of the above definitions were also accepted as some form of understanding of the relevant term - partial understanding and layman terms were also accepted

We included participants who were ≥ 18 years old, able to understand the English language and with capacity to consent. Exclusion criteria were: age < 18 years old, inability to understand the English language and lack of capacity for consent.

## Data collection

Participants were asked to complete the questionnaire (ethical approval reference: 17/NW/0215) on their own and reassured that it was not a test. The questionnaire (S1) had undergone content validation through internal peer review. It recorded participants’ demographics (age, gender, education, occupation, ethnicity, main language spoken, number of children), whether they had been treated for prolapse and understanding of external female anatomy, general medical/gynaecological conditions and POP.

### Anatomical understanding

Anatomical understanding was assessed using two questions:

1. “How many holes does a woman have in her private parts?”
2. A diagram with seven annotated structures (labia majora, labia minora, clitoris, urethra, vagina, perineum, anus) was provided and participants were asked to label as many structures as they could using free text.

### POP understanding

Understanding of POP and general medical/gynaecological conditions, namely diabetes, stroke and fibroids, was assessed. These conditions were included because of their relatively high prevalence in the population and anticipation that the general public would have some knowledge of them. “Fibroids” was chosen as a surrogate for understanding of women’s health conditions, as it is a common gynaecological diagnosis with expected reasonable levels of understanding among the general public. For each condition we asked what the participant understood by these terms (e.g. what do you understand by the word “diabetes”?) followed by free-text space.

The last section included more in-depth questions about POP and participants were asked to proceed only if they had ever heard of any of the following terms: “vaginal prolapse, genital prolapse, prolapse of the womb, pelvic organ prolapse”. Questions included multiple choice questions (e.g. If a woman develops a prolapse, what do you think she should do? (1) Go to an Accident & Emergency department immediately; (2) see a general practitioner (GP) urgently; (3) see a GP at the next routine appointment; (4) do nothing; (5) do not know. There were also open-ended questions followed by free-text spaces, for example “What do you think causes prolapse?”

## Outcome assessment

### Anatomical understanding

Participants were scored out of seven for the number of correct anatomical labels and out of three for correct number of female genital “holes”. Response assessment took into account the use of layman language. For example “peehole” and “bumhole” were accepted as correct labels for the urethra and anus respectively. If either labia majora or minora was labelled correctly, then the participant was labelled as having understood this concept of labia in general.

### POP understanding

POP understanding (in addition to understanding of other diseases) was categorized as ‘any understanding’ versus ‘no understanding’ based on the participant free-text response. Accepted definitions of disease (POP in addition to diabetes, stroke, fibroids) understanding are summarized in Table 1. For blank responses, if at least one other disease question was answered, the response was considered as “no understanding”. If responses to all four disease questions were left blank, this was reported as missing data. “Prolapse” understanding at this part of the questionnaire was analysed separately from the in-depth POP questions asked later in the questionnaire, i.e. a participant could have a poor definition of prolapse at this part but answer some in-depth POP questions correctly, which were judged separately as correct.

## Statistical analysis

Descriptive statistics were used to summarize participant demographics.

### Anatomical understanding

Descriptive statistics were used to identify the proportion of participants identifying the correct number of “holes” and anatomical terms identified in the free-text responses.

The proportion of participants correctly labelling each anatomical structure and the median number of correctly identified genitalia were compared between (1) males and females within the OPD, (2) all OPD and UG and (3) females within the OPD and UG. Significant differences in proportions were assessed via Chi-squared tests and Fisher's exact test where fewer than five participants filled a cell in the contingency tables.

Differences in number of correctly identified labels between these groups were assessed using Mann-Whitney U-tests.

Within females at both clinics, risk factors for increasing correct anatomical labels were assessed via univariable and multivariable linear regression. The following variables were considered: age (18–25, 26–45, 46–65, >65 years), education [≤ secondary, further (college), higher (university)], whether English was a main language, number of children, department attended (OPD, UG), understanding of stroke, diabetes and fibroids (all binary), the number of correctly labelled external female genital structures and previous treatment for POP. All factors were entered into the multivariable model.

A secondary analysis explored ethnicity (white/white British vs. ethnic minorities) as a risk factor for prolapse understanding in a subset of participants who had provided an ethnicity within the questionnaire free text. This variable was tested in univariable and multivariable analyses, the latter adjusting for all other variables in the primary multivariable model.

### POP understanding

The proportions of participants who understood stroke, diabetes, fibroids and prolapse terms were compared using McNemar tests.

Risk factors for understanding prolapse were assessed within females at both clinics using univariable and multivariable logistic regression analyses, including all variables in the previous multivariable model in addition to the number of correct anatomical labels as an additional variable.

In-depth answers for POP-specific questions (causes, symptoms, treatment) in those that had heard of POP were explored descriptively.

## Results

One hundred ninety-one participants returned the questionnaire (31 from UG clinic, 160 from OPD, of which 20 were males). Out of the 171 female participants, 20 (12%) had previously been treated for POP, while 12 (7%) did not know if that was the case. Table 2 summarizes participants’ demographics.

**Table 2:** Participant demographics by gender and department (*n* = 191)

Characteristic	OPD (*N* = 160, 84%)		UG (*N* = 31, 16%)
	Female (*N* = 140, 73%)	Male (*N* = 20, 11%)	Female (*N* = 31, 16%)
Age group			
18–25	35 (18%)	5 (3%)	1 (1%)
26–45	35 (18%)	5 (3%)	10 (5%)
46–65	35 (18%)	5 (3%)	10 (5%)
Over 65	35 (18%)	5 (3%)	10 (5%)
Main language			
English	129 (68%)	20 (10%)	29 (15%)
Other	11 (6%)	0 (0%)	2 (1%)
Ethnicity			
White	68 (36%)	6 (3%)	13 (7%)
Other	69 (36%)	14 (7%)	17 (9%)
Unstated	3 (2%)	0 (0%)	1 (1%)
Education			
Primary	1 (1%)	1 (1%)	0 (0%)
Secondary	39 (20%)	7 (4%)	7 (4%)
Further	44 (23%)	7 (4%)	9 (5%)
Higher	51 (27%)	5 (3%)	14 (7%)
Unstated	5 (3%)	0 (0%)	1 (1%)
Number of children			
0	44 (23%)	8 (4%)	4 (2%)
1	39 (20%)	3 (2%)	8 (4%)
2	28 (15%)	6 (3%)	5 (3%)
3	20 (10%)	0 (0%)	6 (3%)
4	3 (2%)	3 (2%)	6 (3%)
5	2 (1%)	0 (0%)	2 (1%)
6	1 (1%)	0 (0%)	0 (0%)
> 6	3 (2%)	0 (0%)	0 (0%)
Treated for prolapse			
Yes	10 (6%)	N/A	10 (6%)
No	119 (70%)	N/A	20 (12%)
Do not know	11 (6%)	N/A	1 (1%)

*OPD* outpatient department, *UG* urogynaecology clinic

Public understanding of female genital anatomy and pelvic organ prolapse (POP); a questionnaire-based pilot study

### Anatomical understanding

Eighty-eight (46%) participants correctly identified that a female has three “holes” in the external genitalia, which did not differ by department (UG: 52%, OPD 45%, *P* = 0.499). The most commonly mentioned “hole” was the vagina (67%), followed by anus (55%) and then urethra (35%).

For diagram labelling, 88 (46%) were left blank. Out of the 103 who attempted labelling, only 9 (9%) participants labelled all the 7 annotated structures correctly. The top three correctly identified structures were the vagina (71%), anus (67%) and labia (49%). The least correctly identified structure was the perineum (18%).

At the OPD, more females than males correctly identified the vagina (41% and 15% respectively, *P* = 0.028) and anus (39% and 15% respectively, *P* = 0.046), but there was no difference in correct labelling of other structures between genders. The median (IQR) total number of correct labels was higher for OPD females than their male counterparts [1 (0,3) and 0 (0,1) respectively, *P* = 0.022]. There was no difference in correct labelling of any structure between OPD females and UG participants (data not shown).

Different terms were used for urethra, e.g. “peehole”, and anus, e.g. “bumhole” and “back passage”. However, “vagina” was the only term used by participants for the vagina, although with variations in spelling, e.g. ‘virgina’. There was obvious confusion between the urethra and clitoris. Of the 75/103 (73%) participants who labelled clitoris, 47/75 (63%) labelled it correctly but 7/75 (9%) labelled it as the urethra, while 53/103 (51%) labelled the urethra, 27/53 (51%) correctly and 26/53 (49%) as clitoris.

On univariable analysis, the number of correct labels was positively associated with higher education (vs. < secondary), white ethnicity, an understanding of stroke and fibroid (vs. no understanding) and negatively associated with not knowing whether they had had previous POP treatment. On multivariable analysis, however, only higher education (vs. < secondary) (coefficient: 1.05, 95% CI: 0.14, 1.96) and white ethnicity (coefficient: 1.45, 95% CI: 0.58, 2.33) were positively associated with the number of correct labels and not knowing whether they had had previous POP treatment was negatively associated [coefficient: –1.41, 95% CI: −2.70, −0.13)] (Table 3).

**Table 3:** Risk factors for increasing correct labels of external female genitalia among women attending OPD vs. UG clinics in univariable and multivariable analyses

Characteristic	Univariable			Multivariable		
	Coefficient	95% CI	*P* value	Coefficient	95% CI	P value
Sociodemographic						
Age (years)						
18–25	Reference	Reference	Reference	Reference	Reference	Reference
26–45	0.50	−0.40, 1.40	0.272	0.21	−0.71, 1.13	0.648
46–65	−0.06	−0.95, 0.84	0.903	−0.10	−1.06, 0.86	0.833
> 65	−0.63	−1.53, 0.26	0.164	−0.57	−1.62, 0.47	0.280
Education						
≤ Secondary	Reference	Reference	Reference	Reference	Reference	Reference
Further	0.51	−0.24, 1.26	0.178	0.15	−0.71, 1.01	0.733
Higher	1.84	1.12, 2.55	< 0.001	1.05	0.14, 1.96	0.024
English main language	−0.52	−1.74, 0.69	0.398	−0.02	−1.25. 1.20	0.973
Ethnicity (white/white British)*	1.22	0.41, 2.03	0.004	1.45	0.58, 2.33	0.001
Number of children^$^	−0.20	−0.40, 0.01	0.057	−0.10	−0.32, 0.13	0.405
Health literacy						
Understand diabetes	0.32	−0.72, 1.36	0.610	−0.07	−1.13, 0.99	0.891
Understand stroke	1.06	0.35, 1.78	0.004	0.49	−0.27, 1.25	0.205
Understand fibroids	0.95	0.20, 1.69	0.013	0.74	0.00, 1.48	0.050
Clinical department						
General OPD	Reference	Reference	Reference	Reference	Reference	Reference
UG	0.28	−0.52, 1.09	0.491	0.20	−0.66, 1.06	0.639
Previous treatment for POP						
No	Reference	Reference	Reference	Reference	Reference	Reference
Yes	−0.15	−1.10, 0.81	0.759	0.01	−1.04, 1.06	0.985
Do not know	−1.55	−2.75, −0.35	0.012	−1.41	−2.70, −0.13	0.031

OPD: outpatient department, UG: urogynaecology department, POP: pelvic organ prolapse

*Tested in separate models in a subset with available ethnicity data (*n* = 110), with multivariable adjustment for all other factors within this table. ^$^Male participants excluded

Public understanding of female genital anatomy and pelvic organ prolapse (POP); a questionnaire-based pilot study

### POP understanding

Fifty-three per cent of participants understood (or partially understood) POP. A greater proportion understood “diabetes” (87%, *P* < 0.001) and “stroke” (74%, *P* = 0.004) and a lower proportion understood “fibroids” (23%, *P* < 0.001) (Table 4). The most commonly described misunderstanding for POP was confusing the term prolapse with relapse, e.g. drug addiction.

**Table 4:** Relative disease understanding (based on the initial generic disease understanding questions before in-depth POP questions were asked)

Condition	Understood (*N*, %) (*n* = 182)		*P* value comparison with prolapse
	No	Yes	
Prolapse	85 (47)	97 (53)	Reference
Diabetes	23 (13)	159 (87)	< 0.001
Stroke	47 (26)	135 (74)	0.004
Fibroids	141 (77)	41 (23)	< 0.001

POP: pelvic organ prolapse

Public understanding of female genital anatomy and pelvic organ prolapse (POP); a questionnaire-based pilot study

On univariable analysis, increasing age, English as a main language, white ethnicity, increasing number of children and the increasing number of correct anatomical labels, UG department and previous POP treatment were positively associated with increased POP understanding, while further education was associated with decreased POP understanding. Diabetes, stroke or fibroid understanding was not associated with POP understanding.

After multivariable adjustment, older age, white ethnicity and increasing number of correct anatomical labels were significantly associated with POP understanding. Compared with 18–25 years, those 26–45 years had 3.98 times the odds (95% CI 1.22, 13.01, *P* = 0.022), 46–65 years 6.07 times the odds (95% CI 1.77, 20.86, *P* = 0.004) and > 65 years 6.48 times the odds (95% CI 1.67, 25.20, *P* = 0.007) of understanding POP. Compared to non-white, white/white British ethnicity was associated with 4.38 times the odds (CI 1.36, 14.08, *P* = 0.013) POP understanding. For each correct anatomical label the odds of POP understanding increased by 1.43 (CI 1.14, 1.79, *P* = 0.002). Further/higher education, English as a main language, increasing number of children and disease (diabetes, stroke and fibroids) understanding were not associated with POP understanding after multivariable adjustment (Table 5).

**Table 5:** Risk factors for POP understanding among women attending OPD vs. UG clinics in univariable and multivariable analyses

Characteristic	Univariable			Multivariable		
	OR	95% CI	*P* value	OR	95% CI	*P* value
Sociodemographics						
Age (years)						
18–25	Reference	Reference	Reference	Reference	Reference	Reference
26–45	5.45	1.97, 15.09	0.001	3.98	1.22, 13.01	0.022
46–65	7.18	2.53, 20.35	< 0.001	6.07	1.77, 20.86	0.004
> 65	11.68	3.99, 34.19	< 0.001	6.48	1.67, 25.20	0.007
Education						
≤ Secondary	Reference	Reference	Reference	Reference	Reference	Reference
Further	0.41	0.18, 0.96	0.040	0.58	0.19, 1.70	0.318
Higher	0.56	0.25, 1.26	0.161	0.50	0.16, 1.60	0.241
English main language	4.03	1.05, 15.49	0.042	2.84	0.60, 13.42	0.187
Ethnicity (white/white British)*	5.66	2.31,13.86	< 0.001	4.38	1.36, 14.08	0.013
Number of children^$^	1.39	1.10, 1.74	0.005	1.20	0.90, 1.60	0.206
Health literacy						
Understand diabetes	1.40	0.51, 3.84	0.511	1.59	0.40, 6.38	0.511
Understand stroke	1.78	0.87, 3.63	0.114	2.08	0.78, 5.58	0.145
Understand fibroids	1.73	0.81, 3.70	0.156	1.00	0.39, 2.55	0.993
Number of correctly identified anatomy labels	1.28	1.08, 1.51	0.005	1.43	1.14, 1.79	0.002
Clinical department						
General OPD	Reference	Reference	Reference	Reference	Reference	Reference
UG	3.14	1.26, 7.85	0.014	1.29	0.44, 3.80	0.646
Previous treatment for POP						
No	Reference	Reference	Reference	Reference	Reference	Reference
Yes	7.88	1.75, 35.48	0.007	5.00	0.87, 28.59	0.070
Do not know	0.35	0.09, 1.37	0.131	0.74	0.13, 4.07	0.725

OPD: outpatient department, UG: urogynaecology department, POP: pelvic organ prolapse. *Tested in separate models in a subset with available ethnicity data (*n* = 110), with multivariable adjustment for all other factors within this table. ^$^Male participants excluded

Public understanding of female genital anatomy and pelvic organ prolapse (POP); a questionnaire-based pilot study

Ninety-six (50%) participants had heard of at least one POP term and therefore completed the remainder of the questionnaire. Only 35% mentioned bulge symptoms, while 24% listed another symptom without mentioning bulge. Seventy per cent chose ‘no/do not know’ to whether a women who had undergone a previous hysterectomy could develop prolapse; 77% responded that a women who develops a prolapse should seek immediate or urgent medical care (12%: go to A&E immediately; 65%: see GP urgently). The most commonly mentioned treatment was surgery (42%) with 7% mentioning physiotherapy as treatment. Table 6 summarizes participants’ responses to in-depth POP questions.

**Table 6:** Categorisation of participant responses to free-text in-depth POP questions

Question	Response category	*N* (%)
What do you think the symptoms of prolapse are?	Bulge symptoms	34 (35.4)
	Incontinence	14 (14.6)
	Urinary incontinence	8 (8.3)
	Faecal incontinence	1 (1.0)
	Pain	24 (25.0)
	Discomfort	12 (12.5)
	Bleeding	7 (7.3)
	Sex-life disturbance	3 (3.1)
What do you think causes prolapse?	Childbirth	27 (28.1)
	Age	10 (10.4)
	Trauma	7 (7.3)
	Straining	7 (7.3)
	Pregnancy	5 (5.2)
	Overweight	5 (5.2)
	Lifting	3 (3.1)
	Hysterectomy	2 (2.1)
Could a woman who has had a hysterectomy (womb removed) develop prolapse?	Yes	29 (30.2)
	No	25 (26.0)
	Do not know	42 (43.8)
If a woman develops a prolapse, what do you think she should do?*	Go to A&E immediately	11 (11.5)
	See GP urgently	62 (64.6)
	See GP at next routine appointment	19 (19.8)
	Do nothing	0 (0)
	Do not know	4 (4.2)
Do you know of any treatments for prolapse? Please describe	Surgery	40 (41.7)
	Pessary	17 (17.7)
	Pelvic floor exercises	7 (7.3)

*Response options were provided for participants to choose from. POP: pelvic organ prolapse. A&E: Accidents and Emergency, GP: general practitioner. *N*: number of responses

## Discussion

Our study has demonstrated that people attending urogynaecology and general outpatient clinics have poor understanding of female external genital anatomy and prolapse. Women identified more anatomical structures than men; however, the actual number of structures identified was small (median 1 for women and 0 for men). There was obvious confusion between the urethra and clitoris and other common misconceptions included the “cervix” being one of external female genital “holes”.

To our knowledge, this is the first study that explores understanding of female genital anatomy and POP among the general public, both male and female, in the UK. Our study also attempted to confirm the degree of understanding with secondary questions and compared the level of understanding with other medical conditions.

For female external genitalia, just 9% of participants labelled all seven correct, with urethra and clitoris correctly identified by 51% and 63%, respectively. Similarly, a study of 278 women in the USA recruited from a women’s clinic reported correct identification of female external anatomical structures by 67% of participants, with the urethra and clitoris identified by 55% and 57%, respectively [21], which are consistent with our findings. Similar to our findings, the highest identification rates from their study were for the anus (90%) and vagina (84%), albeit our rates were lower, 67% and 71%, respectively. However, their participants were presented with options to choose from to label the diagram. In our study, no options were given; hence, one might expect a lower result and possibly ours represents a more accurate assessment of true knowledge.

We also found far fewer people had an understanding of prolapse compared to the conditions diabetes or stroke. However, fibroids were even less frequently understood, suggesting there is poor knowledge of women’s health issues in general. A study by Weinman et al. in the USA supports this finding. They reported that only 40% of the general public (*n* = 133) correctly identified the site of an ovary on a female body diagram [22]. Another by Lekovic et al. demonstrated that women who had had a previous hysterectomy had similar understanding of physiological functions of female reproductive organs as those who had not, and in both groups, over half of the participants thought pre-menopausal women who have had a total hysterectomy continue to have periods afterwards and that the uterus was the organ producing oestrogen [23]. This is concerning as it implies that women may undergo operations for conditions they do not understand.

We expected that women attending UG would have more understanding of female genital anatomy due to their presenting condition and/or from attending a general practice consultations. However, there was no difference between female OPD and UG participants. This may represent an unmet need of women’s education regarding normal female genital anatomy in general.

Higher education and white ethnicity were positively associated with a greater number of correct anatomical labels. These may reflect other sociodemographic variables, which may lead to exposure to better education at school. This requires further exploration and highlights a particular need for education in particular cross sections of the community.

In our study, 53% understood “prolapse” and 50% had heard of at least one POP term. This is in keeping with other studies in the USA which found that among women attending primary-care gynaecologic clinics (*n* = 376), 52% knew the term “pelvic organ prolapse”, although those were yes/no questions and the participants actual understanding was not confirmed [24]. As with the study of anatomical knowledge, one of the strengths of our study was using free-text responses to test participants understanding of concepts of each disease.

Only 35% of participants mentioned bulge symptoms in relation to POP, while 24% listed another symptom without mentioning bulge. This is an important finding because clinically the only symptom that can be reliably associated with POP is the presence of a vaginal bulge. We found that the majority of women believed that women who develop a prolapse should seek immediate or urgent medical care. This may be due to the misconceptions that a lump could represent cancer, a misconception which Wieslander et al. found in their study [12].

POP understanding was positively associated with increasing age, white ethnicity and increasing number of correct anatomical labels, making the latter the only modifiable factor for POP understanding and showing that efforts to improve health literacy in gynaecology may benefit from targeting younger women using accessible, culturally appropriate methods across diverse populations.

## Limitations

We had planned initially 200 questionnaires to be distributed as part of purposive sampling with even distribution among age groups across both departments. However, in reality the number of 18–25 years old attendees of the UG department was extremely limited, so only one participant was recruited in this group. We suggest future studies to specifically address this age group’s understanding of the topic.

One of the significant limitations of our study is that it is a single-centre study conducted in one country. It does however expand current knowledge from studies conducted in other countries as explained above. However, such studies were confined to women attending gynaecology practices rather than also assessing general outpatients as we did in this study.

This study classified gynaecological anatomy as ‘female’ and assumed that comparisons in health literacy between male and female participants were based on genders assigned at birth. Further work should explore health literacy among transgender people with gynaecological anatomy, who may face additional challenges to shared decision making across multiple clinical settings.

We found 88 diagrams were left blank, and reasons for not labelling the diagram were not ascertained within the questionnaire. Because these were excluded from the analysis, this study may under-estimate the public’s lack of knowledge in this regard.

Another limitation was participant health literacy scores were not formally assessed using validated tools. However, our questionnaire asked about knowledge of common medical conditions for which most people are expected to have some understanding. In our questionnaire, ethnicity was recorded in the form of a free-text question, and participants were to describe their ethnicity the way they felt represented them; however, this made it difficult to then categorize the data since a mixture of ethnicities and nationalities was reported. Thus, we had to exclude some participants who reported on nationalities rather than ethnicities in the primary analysis.

Another limitation was that participants were not supervised while completing their questionnaires. Participants were advised not to discuss answers with others or look up answers and were reassured that the questionnaire was not a test and their responses were confidential. Nevertheless, it is possible that participants could have discussed answers with others or looked them up using electronic devices. Nevertheless, if this occurred then even with access to smart devices, understanding of both female genital anatomy and POP was poor.

## Conclusion

We demonstrated poor public knowledge of external female genital anatomy and understanding of POP in both the UG and OPD settings. Female gender, higher education and white ethnicity were associated with better external female genital anatomy knowledge. POP understanding increased with increasing age, white ethnicity and increased number of correct anatomical labels. Based on these results, the only modifiable factor for such understanding was education/anatomical knowledge.

Future research should focus on effective interventions to breakdown taboos, expand knowledge and thereby empower women to be able to participate in meaningful shared decision making with clinicians.

## Supplementary Information

ESM 1 (DOCX 60 kb)
