# Supplementary material for: Public understanding of female genital anatomy and pelvic organ prolapse (POP); a questionnaire-based pilot study
Source: Int Urogynecol J. 2021 Mar 31;33(2):309–18. doi: 10.1007/s00192-021-04727-9 (PMC8803818; doi:10.1007/s00192-021-04727-9)
Supplement: Supplementary file 1 — (DOCX 60 kb) [file 192_2021_4727_MOESM1_ESM.docx]

Public understanding of female genital anatomy and pelvic organ prolapse (POP); a questionnaire-based pilot study.

# Appendix 1: Study questionnaire

Anonymous Questionnaire on

Public Understanding of Medical Language

We are trying to find out what members of the public understand when we use medical language. This is to try and improve our communication with our patients.

We would be grateful if you could spare a few moments to answer this anonymous questionnaire without your personal details. This is not a test. We want to know what YOU understand. You do not need to use medical language.

When provided with multiple answers, *please circle your responses*.

What do you understand by the word “Diabetes”?

……………………………………………………………………….

………………………………………………………………………..

………………………………………………………………………..

What do you understand by the word “Fibroids”?

……………………………………………………………………….

..................................................................................................

.............................................................................................

What do you understand by the word “Prolapse”?

………………………………………………………………………..

………………………………………………………………………..

………………………………………………………………………..

What do you understand by the word “Stroke”?

………………………………………………………………………..

………………………………………………………………………..

………………………………………………………………………..

We would like to know a little about you:

Where applicable, please circle your response.

How old are you?

18-25 26-45 46-65 above 65

What do you identify your gender as?

Male - Female – Transgender – Prefer not to answer

What education have you had? Please circle all that apply:

Primary – Secondary - Further education (College) - Higher education (University)

What is your occupation?

…………………………………………………………………

Which ethnic group do you consider that you belong to?

…………………………………………………………………

What is the main language you speak at home?

……………………………………………………….………

How many children have you had?

0 - 1 - 2 -3 - 4 - 5 - 6 - more than 6

Have you ever been treated for prolapse before?

Yes – No – Don’t know

Now, we would like to ask you some questions about women’s health: where applicable, please circle your response.

How many holes does a woman have down below (in her private parts)?

1 - 2 - 3 - more than 3 - don’t know

Can you name any of these holes?

………………………………………………………………………

..........................................................................................

..........................................................................................

There is a diagram overleaf for women’s private parts. Please complete as many labels as you know.


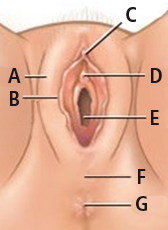


**A**: ………………….. **B**: ………………….

**C**: ………………….. **D**: ………………….

**E**: ………………….. **F**: ………………….

**G**: …………………..

Have you ever heard of any of these conditions (please circle all that apply):

Vaginal prolapse - Genital prolapse - Prolapse of the womb - Pelvic organ prolapse

**If NO**, thank you for your help, you don't need to carry on. **If YES**, please answer the following questions:

What do you think the symptoms of prolapse are?

…………………………………………………………………

…………………………………………………………………

Could women who have had a hysterectomy (womb removed) develop prolapse? Yes - No- Don’t know

If a woman develops a prolapse, what do you think she should do?

Go to A&E immediately - See GP urgently - See GP at next routine appointment - Do nothing - Don't know.

What do you think causes prolapse?

…………………………………………………………………

…………………………………………………………………

…………………………………………………………………

Do you know of any treatments for prolapse? Please describe.

…………………………………………………………………

…………………………………………………………………

…………………………………………………………………

Thank you for taking the trouble to complete this questionnaire.
